# A Rich Hospital

**Published:** 1846-12

**Authors:** 


***A Rich Hospital.***—When shall we have the pleasure of chronicling the first donation or bequest to the "Buffalo City Hospital!" Here is an inviting field for the exercise of philanthropy! Who will take precedence, and thus perpetuate his name by connecting it with an institution which may continue for centuries! What object more purely benevolent can solicit the preference of the wealthy philanthropist! What nobler moment of generosity can be erected!—*Ed. Buffalo Jour.*

***A Rich Hospital.***—The Massachusetts General Hospital and the McLean Lunatic Institution, united in interests, have had the following sums given by the individuals named: John McLean gave $119,000; Mary Belknap, $89,000; Daniel Waldo, $40,000; William Phillips, $25,000; Thos. Oliver, $22,000; Israel Munson, $21,000; Joseph Lee, 20,000; Samuel Illiot, $10,000; Abraham Touro, $10,000; Jeremiah Belknap, $10,000; William Appleton, $12,000; David Sears, $7,000; James Perkins, $5,000; and Mary Brimmer, $5,000.—*Boston Med. and Surg Journal.*

